# Differential Regulation of the Expression of the Two Thyrotropin Beta Subunit Paralogs by Salmon Pituitary Cells *In Vitro*

**DOI:** 10.3389/fendo.2020.603538

**Published:** 2020-11-27

**Authors:** Mitchell Stewart Fleming, Gersende Maugars, Patrick Martin, Sylvie Dufour, Karine Rousseau

**Affiliations:** ^1^ Muséum National d’Histoire Naturelle, Research Unit BOREA, Biology of Aquatic Organisms and Ecosystems, CNRS, IRD, SU, UCN, UA, Paris, France; ^2^ Conservatoire National du Saumon Sauvage (CNSS), Chanteuges, France

**Keywords:** *tshβ* paralogs, *gh*, thyroid hormones, cortisol, IGF1, CRH, *Salmo salar*, pituitary cells *in vitro*

## Abstract

We recently characterized two paralogs of the thyrotropin (TSH) beta subunit in Atlantic salmon, *tshβa* and *tshβb*, issued from teleost-specific whole genome duplication. The transcript expression of *tshβb*, but not of *tshβa*, peaks at the time of smoltification, which revealed a specific involvement of *tshβb* paralog in this metamorphic event. *Tshβa* and *tshβb* are expressed by distinct pituitary cells in salmon, likely related to TSH cells from the *pars distalis* and *pars tuberalis*, respectively, in mammals and birds. The present study aimed at investigating the neuroendocrine and endocrine factors potentially involved in the differential regulation of *tshβa* and *tshβb* paralogs, using primary cultures of Atlantic salmon pituitary cells. The effects of various neurohormones and endocrine factors potentially involved in the control of development, growth, and metabolism were tested. Transcript levels of *tshβa* and *tshβb* were measured by qPCR, as well as those of growth hormone (*gh*), for comparison and validation. Corticotropin-releasing hormone (CRH) stimulated *tshβa* transcript levels in agreement with its potential role in the thyrotropic axis in teleosts, but had no effect on *tshβb* paralog, while it also stimulated *gh* transcript levels. Thyrotropin-releasing hormone (TRH) had no effect on neither *tshβ* paralogs nor *gh*. Somatostatin (SRIH) had no effects on both *tshβ* paralogs, while it exerted a canonical inhibitory effect on *gh* transcript levels. Thyroid hormones [triiodothyronine (T3) and thyroxine (T4)] inhibited transcript levels of both *tshβ* paralogs, as well as *gh*, but with a much stronger effect on *tshβa* than on *tshβb* and *gh.* Conversely, cortisol had a stronger inhibitory effect on *tshβb* than *tshβa*, while no effect on *gh*. Remarkably, insulin-like growth factor 1 (IGF1) dose-dependently stimulated *tshβb* transcript levels, while it had no effect on *tshβa*, and a classical inhibitory effect on *gh*. This study provides the first data on the neuroendocrine factors involved in the differential regulation of the expression of the two *tshβ* paralogs. It suggests that IGF1 may be involved in triggering the expression peak of the *tshβb* paralog at smoltification, thus representing a potential internal signal in the link between body growth and smoltification metamorphosis.

## Highlights

- Atlantic salmon *tshβa* and *tshβb* paralog transcripts are differentially regulated by neuroendocrine factors *in vitro*
- Thyroid hormones have a stronger inhibitory effect on *tshβa* than on *tshβb* expression
- CRH specifically stimulates *tshβa* but not *tshβb* expression, while TRH and SRIH have no effect on both paralogs
-  IGF1 specifically stimulates *tshβb* but not *tshβa* expression
- IGF1 may represent an internal cue linking growth and smoltification

## Introduction

The thyrotropic axis is the major neuroendocrine axis involved in the control of development and metabolism in all vertebrates. It also triggers larval metamorphosis, as investigated in depth in anuran amphibians [for review: (1)], and also shown in some teleosts such as flatfishes [for review: (2)]. Together with other neuroendocrine axes, it may be involved in the control of other life history transitions, such as smoltification (parr-smolt transformation) in salmonids. Smoltification is a crucial late developmental event, which triggers the downstream migration to the sea of the juvenile salmon and preadapts it to seawater conditions, where it will complete its oceanic growth phase before returning to its natal river to spawn [for reviews: (3–5)]. Smoltification is referred to as “secondary metamorphosis” by some authors, by comparison to the “primary” or “true” larval metamorphosis [for reviews: (6–8)].

Classically, in mammals, the thyrotropic axis comprises a cerebral neuropeptide named thyrotropin-releasing hormone (TRH), which acts on the pituitary to induce the synthesis and release of thyrotropin (thyroid-stimulating hormone; TSH). This pituitary hormone stimulates the production by the thyroid gland of thyroid hormones, thyroxine (T4) and triiodothyronine (T3), which act on a variety of peripheral target organs. The thyroid hormones also exert negative feedbacks on the brain and pituitary to regulate TSH production [for reviews: (9, 10)]. In non-mammalian vertebrates (amphibians and birds), some variations are observed in the thyrotropic axis, notably concerning the central control of TSH, which can also involve corticotropin-releasing hormone (CRH) [for review: (11)], a neuropeptide initially discovered in mammals for its role in the control of pituitary corticotropin and stress axis [for reviews: (12, 13)].

TSH, like gonadotropins, LH, and FSH, is a pituitary glycoprotein composed of two subunits, the alpha subunit (glycoprotein hormone alpha subunit, gp*a*), in common with gonadotropins, and a specific beta subunit (*tshβ*), which confers the hormone specificity [for review: (14)]. Due to teleost-specific whole genome duplication [TSWGD; also referred to as “3R,” for “third round of whole genome duplication” (15)], teleosts possess two *tshβ* paralogs, *tshβa* and *tshβb* (16).

Our recent studies in Atlantic salmon, *Salmo salar*, revealed a remarkable functional divergence of the duplicated *tshβ* paralogs, with a striking peak of pituitary *tshβb* transcripts at the time of smoltification, in early April, concomitantly with the change in rheotaxism and initiation of downstream migration, whereas no change was observed for *tshβa*. While previous studies by many authors in salmonids had only concerned the “classical” *tshβa* paralog, the demonstration of another paralog and its expression peak at smoltification provided the first evidence for the involvement of TSH in smoltification metamorphosis (17).

Furthermore, as shown by *in situ* hybridization, the two Atlantic salmon *tshβ* paralogs are expressed by two different cell populations in the pituitary: *tshβa* cells are abundant and located in the anterior adenohypophysis, while *tshβb* cells are less numerous, well detected only at the time of smoltification, and located in the dorsal adenohypophysis near to the pituitary stalk (17). This differential localization and abundance of TSH cells in salmon could be compared to the situation in mammals and birds, which present a “classical” TSH cell population in the pituitary *pars distalis* (PD) and a less numerous TSH cell population in the *pars tuberalis* (PT) adjacent to the pituitary stalk [mammals (18–20); birds (21)]. In birds and mammals, PT-TSH acts retrogradely on hypothalamic area and is involved in the brain regulation of seasonal life-traits, such as reproduction, migration and hibernation [for reviews: (22–24)]. A similar potential role in the seasonal regulation of smoltification could be hypothesized for the *tshβb* paralog in salmon (17). While in birds and mammals, both pituitary TSH cell populations express the same single *tshβ* gene, the two TSH cell populations in Atlantic salmon express distinct *tshβa* and *tshβb* paralogs. This differential localization of the two paralogs, together with their sequence divergence [33% identity; 48% similarity; (17)] and their striking differential regulation at smoltification, illustrate a typical case of subfunctionalization (17).

The present study aims at investigating whether the expression of the two *tshβa* and *tshβb* paralogs are under differential central and peripheral controls. As for our previous investigations (17), this study was performed on Atlantic salmon from the Loire-Allier population, the last extant population from long-river in Western Europe, currently endangered, and under a conservation program at the Conservatoire National du Saumon Sauvage (CNSS). The regulation and timing of smoltification is an especially critical issue for this long-river population, as compared to short-river-ones, as smoltification occurs up to 900 km upstream, and smolts need to perform a long downstream migration and reach the estuary in a narrow window of favorable physiological and environmental conditions.

We used primary cultures of Atlantic salmon pituitary cells to study the direct pituitary control of the expression of both *tshβ* genes. While in birds and mammals, the same *tshβ* gene is expressed in both PD and PT cells, here we take advantage of the expression of distinct paralogs, *tshβa* and *tshβb*, by the two TSH cell populations in salmon, which enables us to follow their differential expression in whole mixed pituitary cell cultures. We tested the effects of central neurohormones, known to be involved in TSH regulation in vertebrates (TRH, CRH, and somatostatin, SRIH) as well as peripheral hormones reported to increase before and/or during smoltification [thyroid hormones, insulin-like growth factor (IGF1), and cortisol] [for reviews: (6, 25)]. For comparison and validation, we also followed the regulation of the expression of growth hormone, *gh*, which may share some common regulatory factors with TSH in vertebrates, and which is involved in the regulation of osmoregulatory changes at smoltification in salmonids [for review: (26)].

## Material and Methods

### Animals

Atlantic salmon (*Salmo salar*) from the Loire-Allier population raised indoor under natural water, temperature, and photoperiod conditions, at the Conservatoire National du Saumon Sauvage (CNSS), Chanteuges, France (Agreement N° B43 056 005; according to the ARRETE N° DDCSPP/CS/2016/40), were used. They were anesthetized with an overdose of ms222 (0.4 ml/L; Sigma-Aldrich, St. Louis, MI, USA) and killed by decapitation in accordance with guidelines and regulations according to the protocol approved by Cuvier Ethic Committee, France. For each cell culture, pituitaries from 40 to 100 juvenile salmon were collected in cell culture medium at CNSS, and immediately transferred on ice to MNHN, Paris, where cell dispersion and cultures were performed. In order to be able to detect *tshβb* in pituitary cell cultures, salmon pituitaries were collected at different times in April (years 2015 and 2018) during the peak of its expression (17). For *tshβa* expression data, some additional experiments have been performed from January to April (years 2015, 2016, and 2018) with similar results as in April.

### Hormones

Thyroid hormones and cortisol were purchased from Sigma-Aldrich (Saint-Quentin Fallavier, France; triiodothyronine, T3, catalog number: T2752; thyroxine, T4, catalog number: T2501; cortisol, F, catalog number: H4001), and used at similar doses as in previous *in vitro* studies in the eel (27, 28), covering circulating ranges.

Bovine corticotropin-releasing hormone (CRH), which was shown to be effective on primary culture of pituitary cells from other teleost species [eel: (29); turbot: (30)], was purchased from Sigma-Aldrich (catalog number: C2671), and used at similar doses as in previous studies.

Thyrotropin-releasing hormone (TRH; catalog number: P1319) and somatostatin (SRIH; catalog number: S1763), fully conserved in vertebrates, were purchased from Sigma-Aldrich, and used at similar doses as in previous *in vitro* studies in other teleost species [eel: (29, 31); turbot: (30)].

Recombinant human insulin-like growth factor 1 (IGF1), which was shown to be effective on primary culture of pituitary cells from other teleost species [eel: (32); turbot: (33)], was purchased from R and D Systems (Lille, France; catalog number: 291-G1), and used at similar doses as in previous studies.

As in our previous studies, T3 and T4 were dissolved in NaOH 1N, cortisol in ethanol, CRH, TRH and SRIH in sterile water and IGF1 in sterile PBS to obtain stock solutions (10^-3^ M), which were stored at −20°C.

### Primary Culture of Salmon Pituitary Cells

#### Dispersion and Culture

Dispersion and primary culture of pituitary cells were performed using an enzymatic and mechanical method adapted from [eel (34); salmon (35)]. Briefly, 40 to 100 pituitaries were incubated at 25°C in a solution of 0.8 mg porcine type II trypsin (Sigma-Aldrich)/ml dispersion buffer (DB: Dulbecco’s saline phosphate buffer without Ca^2+^ and Mg^2+^, with 100 U/ml penicillin, 100 µg/ml streptomycin, and 250 ng/ml fungizone; Gibco, Thermo Fisher Scientific, Villebon-sur-Yvette, France). After 30 min, the trypsin solution was replaced by a solution of 1 μg DNase (Sigma-Aldrich) and 1 mg soya bean trypsin inhibitor (Sigma-Aldrich)/ml DB for 10 min. Pituitary slices were then washed with DB (Gibco) and mechanically dispersed in DB by repeated passages through a plastic transfer pipette (Falcon, Thermo Fisher Scientific). Cell suspensions were filtered through nylon mesh (30 μm pore size), harvested by centrifugation at 200 g for 10 min, resuspended in DB, and counted with a Malassez cytometer. The number of viable cells was estimated by Trypan Blue coloration exclusion (Sigma-Aldrich) and was about 90%. Cells were plated on 96-well plates (~60,000 cells/well) pre-coated with a solution of 0.1 mg/ml poly-L-lysine (Sigma-Aldrich). Cultures were performed in serum-free culture medium (Medium 199 with Earle’s salt and sodium bicarbonate buffer, 100 U/ml penicillin, 100 µg/ml streptomycin, and 250 ng/ml fungizone; Gibco) at 18°C under 3% CO_2_ and saturated humidity in tissue culture incubator (Galaxy 170R, Eppendorf, France).

#### In Vitro Treatments

Treatments were started 24 h after the beginning of culture to allow cell attachment (Day 0). Replicates of five wells for control and each treated group were used. Stock solutions (10^-3^M) were diluted in culture medium just before addition to the culture wells. Culture medium was changed and treatment added to the cells on Day 0, Day 3, and Day 7. Cultures were stopped on Day 10, according to our previous protocol (36–38). The effects of treatments were tested in three to five independent experiments performed on different cell preparations from different batches of fish. Figures display the results of representative experiments.

#### Cell RNA Extraction and cDNA Synthesis

Total RNA was directly extracted from cells in culture wells using the Cell-to-cDNA II Kit (Ambion Inc. Austin, TX, USA) according to the manufacturer’s recommendations. Cells were washed with PBS (Gibco) and lysed with Cell Lysis II Buffer (80 µl/well). The lysates were digested with RNase-free DNase I (Roche Ltd., Basel, Switzerland). Four µl of RNA solution of each samples was then reverse transcribed with the SuperScript III First Strand cDNA Synthesis Kit (Invitrogen Cergy-Pontoise, France). The samples obtained were stored at −20°C until qPCR.

### Real-Time Quantitative PCR (qPCR)

Gene specific primers were previously designed based on the nucleotide sequences of the Atlantic salmon thyrotropin-*β* subunit paralog a and paralog b (*tshβa* and *tshβb*) and *β-actin* (17), the latter being used as reference gene (**Table 1**). For growth hormone, *gh*, as the two paralogs, *gh1* and *gh2*, issued from salmonid-specific WGD (40) have highly similar sequences and were regulated in the same manner in our pilot experiments (as tested on pooled samples, data not shown), common *gh* specific primers (**Table 1**) were designed for Atlantic salmon, using the Primer3 Software (Whitehead Institute/Massachusetts Institute of Technology, Boston, MA, USA). Forward and reverse primers were located in different exons to prevent amplification of genomic DNA. To optimize the assay, different annealing temperatures were tested according to the melting temperature (Tm) of primers. To check their specificity, amplification products were sequenced at GATC Biotech (Mulhouse, France).

**Table 1:** Primer sequences used in qPCR amplifications.

Primers	5’-3’ sequence (bp)	
*actin*-F	CCAAAGCCAACAGGGAGAAG	Olsvik et al. (39)
*actin*-R	AGGGACAACACTGCCTGGAT	Fleming et al. (17)
*tshβa*-F	CTCCTTTGCCTGCTCTTCAG	Fleming et al. (17)
*tshβa*-R	GGCCAGCTCCTTCATGTTAC	Fleming et al. (17)
*tshβb*-F	TTGCCGTCAACACCACCAT	Fleming et al. (17)
*tshβb*-R	GGGATGATAGACCAGGGAGTG	Fleming et al. (17)
*gh*-F	AGAAGCTCAGCGACCTCAAA	This study
*gh*-R	TGTCATCCAGGCTCAGTACG	This study

The table provides the sequences of forward (F) and reverse (R) primers used for qPCR of Atlantic salmon β-actin, tshβa, tshβb, and gh.

Quantitative PCR assays were performed using the LightCycler^®^ System (Roche) with SYBR Green I sequence-unspecific detection as previously described (17, 38). The qPCRs were prepared with 4 µl of diluted cDNA template, 2 µl of PCR grade water, 2 µl of SYBR Green master mix, and 1 µl of each forward and reverse primer (500 nM each at final concentration). The protocol was an initial step of polymerase activation for 10 min at 95°C; then 41 cycles (*β-actin*, *gh* and *tshβa*) of 10 s at 95°C for denaturing, 5 s at 60°C for annealing, 10 s at 72°C for primer extension, and a single final extension step of 5 min at 72°C. For *tshβb*, the protocol was an initial step of polymerase activation for 10 min at 95°C; 51 cycles of 10 s at 95°C, 5 s at 62°C, 10 s at 72°C, and a single final extension step of 5 min at 72°C. Each program ended with a melting curve analysis by slowly increasing the temperature (0.01°C/s) up to 95°C with a continuous registration of changes in fluorescent emission intensity. Serial dilutions of cDNA pools of pituitary cells were used as a standard curve. One chosen dilution was also included in each run as a calibrator. Each qPCR run contained a non-template control (cDNA was substituted by water) for each primer pairs to confirm that reagents were not contaminated. The efficiency of all primers was tested, and the specificity of each reaction was assessed by melting curve analysis to ensure the presence of only one product. Each sample was analyzed in duplicate by qPCR. Normalization of data was performed using *β-actin* mRNA level and results expressed as arbitrary units, relatively to mean value of control group, considered as 1.

### Statistics

Results are given as mean ± SEM (n = 5 wells/treatment; 60,000 cells/well; cell culture from 40–100 pituitaries). Means were compared by one-way ANOVA Tukey’s multiple comparison test using Instat (GraphPad Software Inc., San Diego, CA, USA). Differences are considered significant when P < 0.05.

## Results

### Detection of *tshβa* and *tshβb* Transcripts by qPCR in Pituitary Cell Cultures

Transcript levels of *tshβa* paralog could be well detected by qPCR in primary cultures of pituitary cells (~60,000 cells/well) with similar expression levels in juvenile Atlantic parr or smolt, as tested in January (parr), April (smolt), and June (post-smolt) (data not shown). In contrast, transcript levels of *tshβb* paralog in cell cultures were under qPCR detection limit in January and June and could be measured in April. In April, basal expression levels of *tshβb* were still lower than that of *tshβa*, as suggested by the difference in the average quantification cycle values (Cq) (20 Cq for *tshβa* and 28 Cq for *tshβb*, which would correspond to about 500-fold difference in abundance).

### Effects of Thyroid Hormones (T3 and T4)

**Figure 1:**
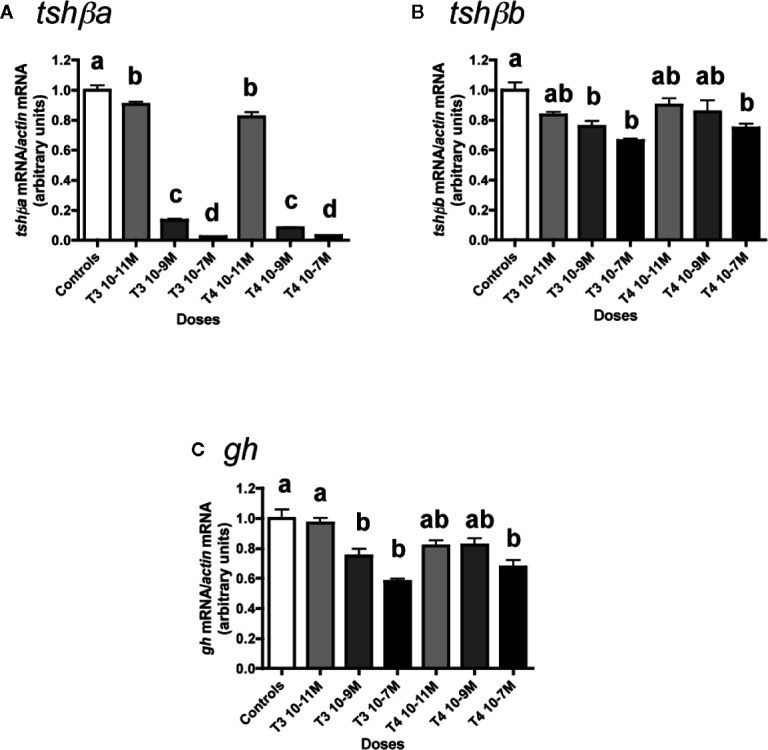
Effects of thyroid hormones (T3 and T4) on *tshβa*, *tshβb*, and *gh* transcript levels in primary cultures of Atlantic salmon pituitary cells. Pituitary cells from juvenile Atlantic salmons were treated with various concentrations of T3 or T4 for 10 days. The mRNA levels of *tshβa* **(A)**, *tshβb* **(B)**, and *gh* **(C)** were quantified by qPCR. Data were normalized against *β-actin*. The Figure displays the results from a representative experiment of three (*tshβb*) or five (*tshβa* and *gh*) independent cell culture experiments. Mean ± SEM; n = 5 well replicates. Different letters indicate significant differences, ANOVA.

The effects of various concentrations of T3 and T4 (from 10**^-^** ^11^ to 10**^-^** ^7^ M) were tested (**Figure 1**). Both T4 and T3 dose-dependently downregulated *tshβa* mRNA levels (**Figure 1A**). Their effects were significant at 10**^-^** ^11^ M (10% inhibition as compared to controls, P < 0.05 for T3 and 18% inhibition, P < 0.001 for T4) and reached for both hormones more than 85% inhibition at 10**^-^** ^9^ M (P < 0.001) and more than 95% inhibition at 10**^-^** ^7^ M (P < 0.001).

T3 and T4 also dose-dependently decreased *tshβb* mRNA levels, but with a lower inhibitory effect than on *tshβa* (**Figure 1B**). For T3, the effect was significant at 10**^-^** ^9^ M (24% inhibition, P < 0.01) and reached 34% inhibition at 10^-7^ M, as compared to controls. For T4, the effect was significant at 10^-7^ M with 25% inhibition.

T3 and T4 dose-dependently inhibited *gh* expression (**Figure 1C**). For T3, the effect was significant at 10**^-^** ^9^ M (25% inhibition, P < 0.01) and reached 42% inhibition at 10**^-^** ^7^ M (P < 0.001) as compared to controls. For T4, the effect was significant at 10^-7^ M with 32% inhibition (P < 0.001).

### Effects of Cortisol (F)

**Figure 2:**
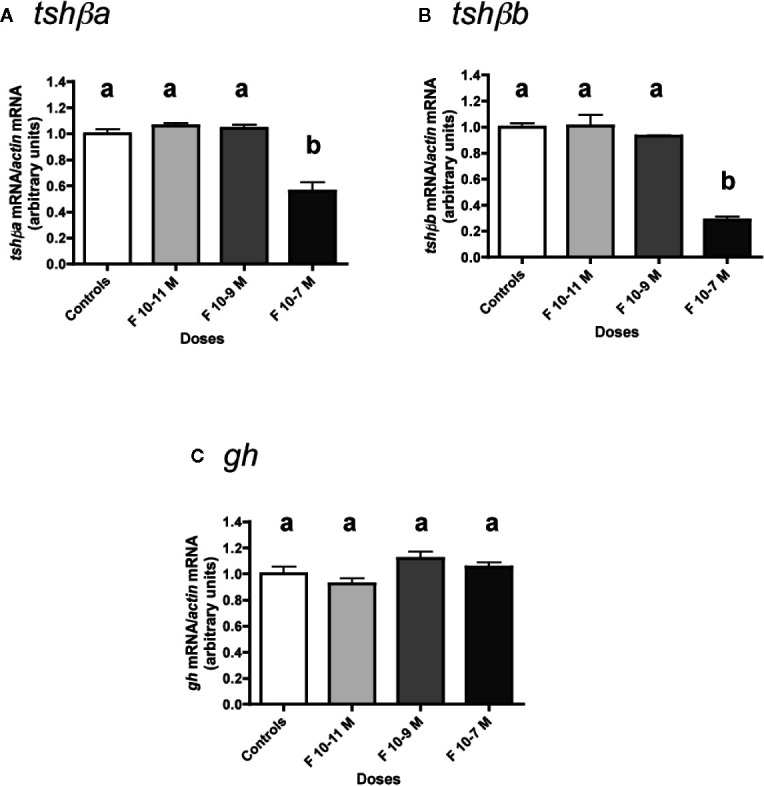
Effects of cortisol (F) on the expression of *tshβa*, *tshβb*, and *gh* transcript levels in primary cultures of Atlantic salmon pituitary cells. Pituitary cells from juvenile Atlantic salmons were treated with various concentrations of F for 10 days. The mRNA levels of *tshβa* **(A)**, *tshβb* **(B)**, and *gh* **(C)** were quantified by qPCR. Data were normalized against *β-actin*. The Figure displays the results from a representative experiment of three independent cell culture experiments. Mean ± SEM; n = 5 well replicates. Different letters indicate significant differences, ANOVA.

The effects of various concentrations of F (from 10**^-^** ^11^ to 10**^-^** ^7^ M) were tested (**Figure 2**).

Low doses (10^-11^ and 10^-9^ M) of F were ineffective in regulating *tshβa* mRNA levels, but 10^-7^ M had a significant inhibitory effect (44% inhibition; P < 0.001, as compared to controls) (**Figure 2A**).

Low doses (10^-11^ and 10^-9^ M) of F had no effect on *tshβb* mRNA levels, while 10^-7^ M was able to strongly reduce them (72% inhibition; P < 0.001) (**Figure 2B**).

In contrast, none of the doses of F tested had any effect on *gh* mRNA levels (**Figure 2C**).

### Effects of CRH and TRH Alone

**Figure 3:**
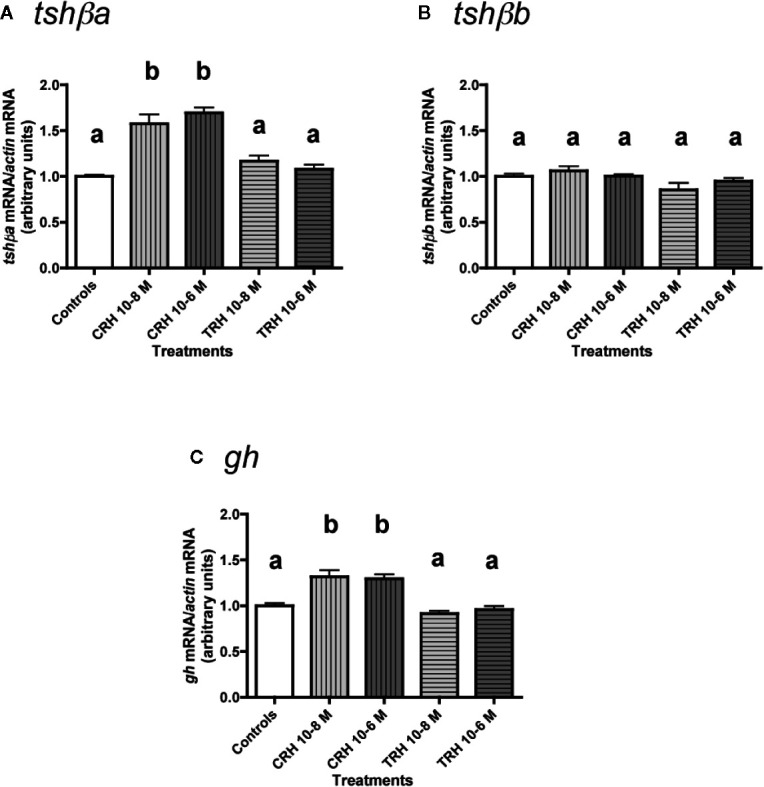
Effects of CRH and TRH on *tshβa*, *tshβb*, and *gh* transcript levels in primary cultures of Atlantic salmon pituitary cells. Pituitary cells from juvenile Atlantic salmons were treated with 10^-8^ and 10^-6^ M of CRH or TRH for 10 days. The mRNA levels of *tshβa* **(A)**, *tshβb* **(B)**, and *gh* **(C)** were quantified by qPCR. Data were normalized against *β-actin*. The Figure displays the results from a representative experiment of three independent cell culture experiments. Mean ± SEM; n = 5 well replicates. Different letters indicate significant differences, ANOVA.

The effects of CRH and TRH (10^-8^ and 10^-6^ M) were tested (**Figure 3**). Both doses of CRH significantly increased the mRNA levels of *tshβa* (×1.6 for 10^-8^ M; ×1.7 for 10^-6^ M; P < 0.001 for both doses, as compared to controls) (**Figure 3A**). In contrast, TRH had no effect on *tshβa* mRNA levels at both doses (**Figure 3A**).

None of the two tested doses of CRH and TRH had any effect on *tshβb* mRNA levels (**Figure 3B**).

Both doses of CRH increased the mRNA levels of *gh* (×1.3; P < 0.001 for both doses) (**Figure 3C**). TRH had no effect on *gh* mRNA levels at both doses (**Figure 3C**).

### Effects of CRH and TRH in the Presence of T3

**Figure 4:**
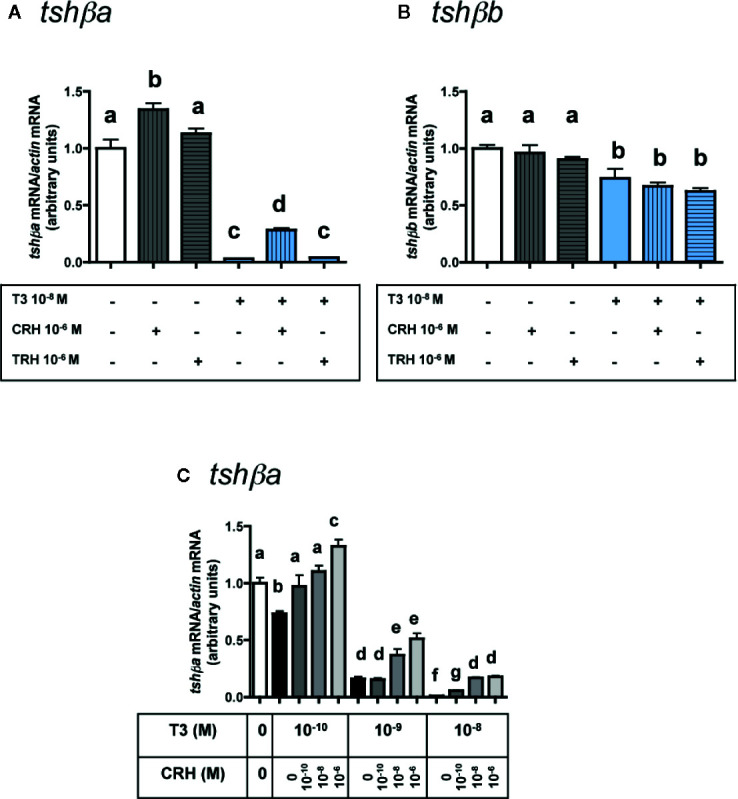
Effects of CRH and TRH on *tshβa* and *tshβb* transcript levels in the presence of T3 in primary cultures of Atlantic salmon pituitary cells. Pituitary cells from juvenile Atlantic salmons were treated for 10 days with 10^-6^ M of CRH or 10^-6^ M TRH in the presence of 10^-8^ M of T3, and mRNA levels of *tshβa* **(A)** and *tshβb* **(B)** were quantified by qPCR. In **(C)**, pituitary cells were treated for 10 days with various doses of CRH (10^-10^, 10^-8^, and 10^-6^ M) in the presence of various doses of T3 (10^-10^, 10^-9^, and 10^-8^ M) and mRNA levels of *tshβa* were quantified by qPCR. The Figure displays the results from a representative experiment of three independent cell culture experiments. Mean ± SEM; n = 5 well replicates. Different letters indicate significant differences, ANOVA.

The potential stimulatory effects of CRH or TRH on *tshβ* paralogs were further investigated by testing the effects of CRH and TRH in combination with the inhibitory effect of T3 (**Figure 4**).

As observed above (**Figure 3A**), 10^-6^ M CRH alone stimulated *tshβa* mRNA levels (×1.3; P < 0.001), while 10^-6^ M TRH alone had no effect (**Figure 4A**). T3 at 10^-8^ M, as observed in **Figure 1C**, inhibited *tshβa* mRNA levels by more than 90% inhibition (P < 0.001) (**Figure 4A**). In the presence of 10^-8^ M T3, the stimulation of *tshβa* mRNA levels by 10^-6^ M CRH was greater (×8.8; P < 0.001) than when tested alone, while 10^-6^ M TRH had still no effect (**Figure 4A**).

Concerning the *tshβb* paralog, as observed above (**Figure 3B**), 10^-6^ M of CRH or TRH alone had no effect on *tshβb* mRNA levels (**Figure 4B**). 10^-8^ M T3, as observed in **Figure 1C**, inhibited *tshβb* mRNA levels (26% inhibition) (**Figure 4B**). In the presence of T3, 10^-6^ M of CRH and TRH had still no effect on *tshβb* mRNA levels (**Figure 4B**).

In order to further assess the stimulatory effect of CRH on *tshβa* mRNA levels, different doses of CRH (10^-10^, 10^-8^, and 10^-6^ M) were tested in the presence of different doses of T3 (10^-10^, 10^-9^, and 10^-8^ M) (**Figure 4C**). T3 (10^-10^ M) inhibited *tshβa* mRNA levels by 27% compared to controls (P < 0.001). CRH dose-dependently stimulated *tshβa* mRNA levels in the presence of 10^-10^ M T3 (up to ×1.8 at 10^-6^ M; P < 0.001, as compared to 10^-10^ M T3 alone), which reached values higher than in non-treated controls (×1.3; P < 0.001; as compared to controls). T3 (10^-9^ M) inhibited *tshβa* mRNA levels by 84% compared to controls (P < 0.001). CRH dose-dependently stimulated *tshβa* expression in the presence of 10^-9^ M T3 (up to ×3.2 at 10^-6^ M; P < 0.001, as compared to 10^-9^ M T3 alone). T3 at 10^-8^ M inhibited *tshβa* mRNA levels by more than 90% compared to controls (P < 0.001). CRH dose-dependently stimulated *tshβa* mRNA levels in the presence of 10^-8^ M T3 (up to ×17 at 10^-6^ M; P < 0.001, as compared to 10^-8^ M T3 alone), but which still remained lower than in non-treated controls. Thus, the stimulatory effect of CRH on *tshβa* was largely enhanced in the presence of high inhibitory doses of T3.

### Effects of CRH and TRH in the Presence of F

**Figure 5:**
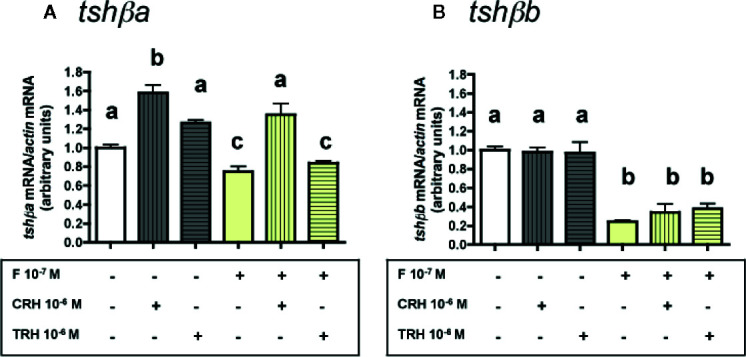
Effects of CRH and TRH on *tshβa* and *tshβb* transcript levels in the presence of cortisol in primary cultures of Atlantic salmon pituitary cells. Pituitary cells from juvenile Atlantic salmons were treated for 10 days with 10^-6^ M of CRH or 10^-6^ M TRH in the presence of 10^-8^ M of cortisol (F) and mRNA levels of *tshβa* **(A)** and *tshβb* **(B)** were quantified by qPCR. Data were normalized against *β-actin*. The Figure displays the results from a representative experiment of three independent cell culture experiments. Mean ± SEM; n = 5 well replicates. Different letters indicate significant differences, ANOVA.

Similarly, the potential stimulatory effects of CRH (10^-6^ M) or TRH (10^-6^ M) were tested in combination with the inhibitory effect of 10^-7^ M F (**Figure 5**).

As observed above (**Figures 3A** and **4A**), 10^-6^ M CRH alone stimulated *tshβa* mRNA levels (×1.6; P < 0.001), while 10^-6^ M TRH had no effect. F at 10^-7^ M, as observed in **Figure 2A**, moderately inhibited *tshβa* mRNA levels (25% inhibition). In the presence of F, the stimulation of *tshβa* mRNA levels by 10^-6^ M CRH was also found (×1.8; P < 0.001, as compared to 10^-7^ M F alone), while 10^-6^ M TRH had no effect (**Figure 5A**).

As observed above (**Figures 3B** and **4B**), 10^-6^ M of CRH or TRH alone had no effect on *tshβb* mRNA levels. F at 10^-7^ M, as shown in **Figure 2B**, strongly inhibited *tshβb* mRNA levels (76% inhibition). In the presence of F, 10^-6^ M of CRH and TRH had still no effect on *tshβb* mRNA levels (**Figure 5B**).

### Effects of SRIH

**Figure 6:**
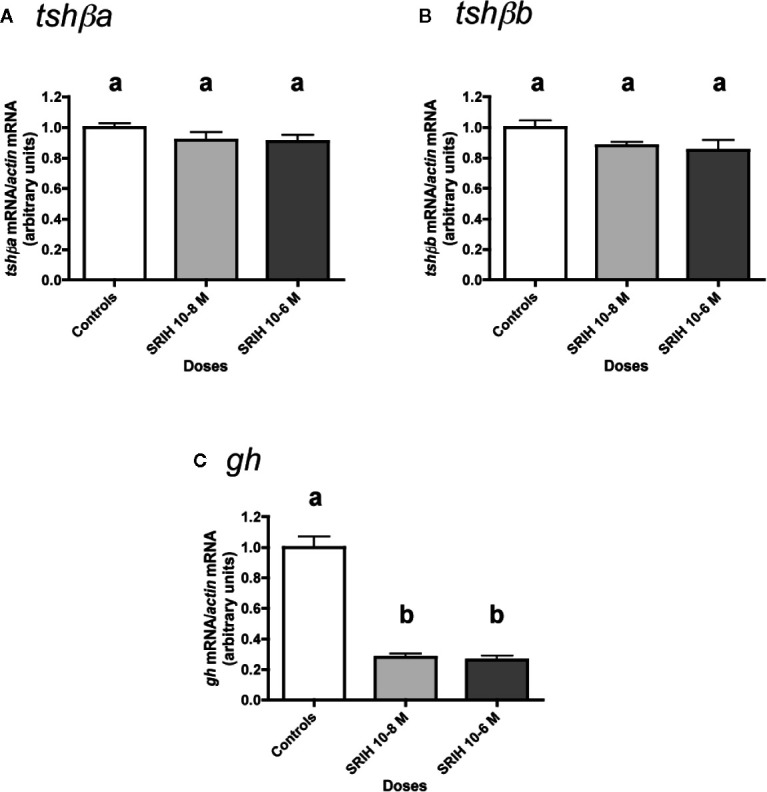
Effects of SRIH on *tshβa*, *tshβb*, and *gh* transcript levels in primary cultures of Atlantic salmon pituitary cells. Pituitary cells from juvenile Atlantic salmons were treated with 10^-8^ and 10^-6^ M of SRIH for 10 days. The mRNA levels of *tshβa* **(A)**, *tshβb* **(B)**, and *gh* **(C)** were quantified by qPCR. Data were normalized against *β-actin*. The Figure displays the results from a representative experiment of three independent cell culture experiments. Mean ± SEM; n = 5 well replicates. Different letters indicate significant differences, ANOVA.

The effect of SRIH (10^-8^ and 10^-6^ M) was tested (**Figure 6**). None of the two tested doses of SRIH had any effect on *tshβa* and *b* mRNA levels (**Figures 6A, B**). In contrast, *gh* mRNA levels were strongly downregulated by SRIH (72% inhibition at 10^-8^ M and 74% inhibition at 10^-6^ M; P < 0.001 for both doses, as compared to controls) (**Figure 6C**).

### Effects of IGF1

**Figure 7:**
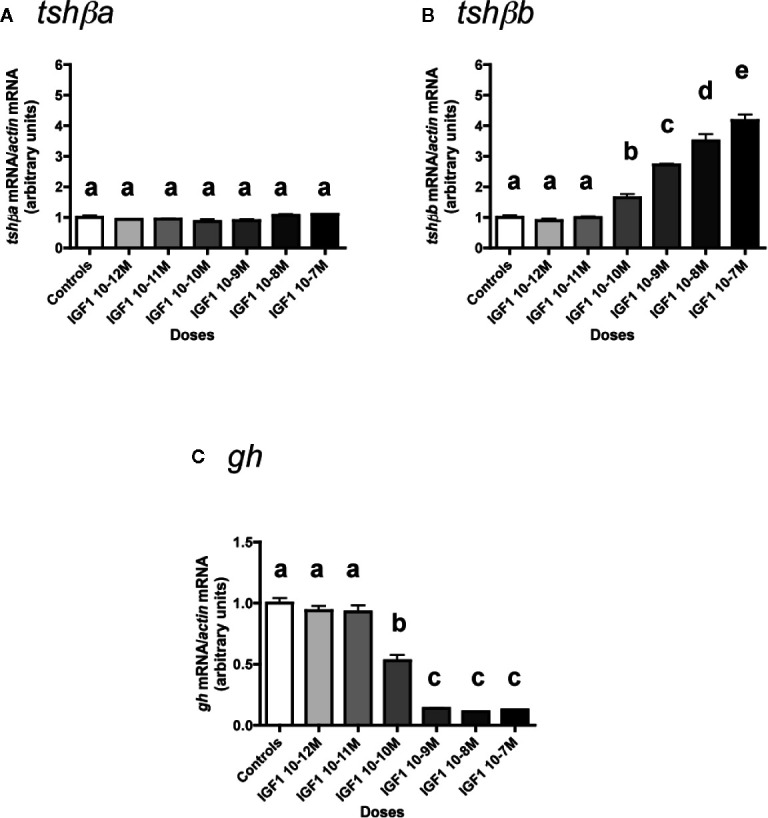
Effects of IGF1 on *tshβa*, *tshβb* and *gh* transcript levels in primary cultures of Atlantic salmon pituitary cells. Pituitary cells from juvenile Atlantic salmons were treated with various concentrations of IGF1 for 10 days. The mRNA levels of *tshβa* **(A)**, *tshβb* **(B)**, and *gh* **(C)** were quantified by qPCR. Data were normalized against *β-actin*. The Figure displays the results from a representative experiment of three independent cell culture experiments. Mean ± SEM; n = 5 well replicates. Different letters indicate significant differences, ANOVA.

The effects of various concentrations of IGF1 (from 10**^-^** ^12^ to 10**^-^** ^7^ M) were tested (**Figure 7**). None of the six tested doses of IGF1 had any significant effect on *tshβa* mRNA levels (**Figure 7A**).

In contrast, IGF1 dose-dependently and strongly stimulated mRNA levels of the other paralog, *tshβb* (**Figure 7B**). The stimulatory effect of IGF1 was significant from 10^-10^ M (×1.6; P < 0.05, as compared to controls) and reached ×4.2 at 10^-7^ M (P < 0.001, as compared to controls).

On the opposite*, gh* mRNA levels were downregulated in a dose-dependent manner by IGF1 (**Figure 7C**). The inhibitory effect of IGF1 was significant at 10^-10^ M (47% inhibition) and reached a plateau with more than 85% inhibition at 10^-9^ M (P < 0.001 for all doses).

## Discussion

In the present study, we investigated the differential regulation of the duplicated *tshβa* and *tshβb* paralogs in the juvenile Atlantic salmon by various central and peripheral neuroendocrine factors. We were especially interested in deciphering whether one of these factors could be potentially involved in the regulation of the striking peak of *tshβb* transcript expression observed at the time of smoltification (17).

### Expression of *tshβa* and *tshβb* Paralogs in Primary Cultures of Atlantic Salmon Pituitary Cells

We investigated the regulatory effects of neurohormones and hormones, as exerted directly at the pituitary level, by using primary cultures of Atlantic salmon pituitary cells. In mammals and birds, the same single *tshβ* gene is expressed by the two distinct pituitary PD- and PT-TSH cell populations, that requires to micro-dissect the PT and PD pituitary regions in order to investigate potential differential regulations in cell cultures. A series of investigations have been performed on primary cultures of ovine PT cells (41–44), but to our knowledge, these studies did not address the regulation of *tshβ* expression. Here, we took advantage of the *tshβa* and *tshβb* paralogs issued from the teleost whole genome duplication, each one being expressed in distinct anterior and dorsal pituitary TSH cell populations in Atlantic salmon. This makes it possible to evaluate their differential transcriptional regulation in the same experiments and the same conditions, in primary cultures of mixed pituitary cells.

Our culture system is based on a low number of pituitary cells per well (~60,000 cells/well), as previously developed for the eel, *Anguilla anguilla* (31, 32), in order to limit the number of fish being used, in agreement with the international recommendations for animal experimentation (Reduce, Refine, Replace), and especially relevant for species concerned by biodiversity conservation. In these conditions, both *tshβa* and *tshβb* transcripts could be measured by qPCR in primary cultures of pituitary cells of Atlantic salmon smolts, as performed in April at the time of smoltification climax. In contrast, *tshβb* mRNA levels were under qPCR detection limit in pituitary cell cultures from parr (January) and post-smolts (June). This is in agreement with our previous studies on the annual expression profiles of *tshβa* and *tshβb* paralogs *in vivo*, as measured by qPCR on whole pituitaries. *In vivo*, *tshβa* pituitary transcript levels remained unchanged from December to June, including the period of smoltification. In contrast, *tshβb* transcript levels were very low in juvenile salmon parr, in December and January, started increasing in February–March to reach a peak in early April at the time of smoltification-related rheotaxism inversion, and dropped in May to reach back very low levels in June in post-smolts (17). Similarly, the lower abundance of *tshβb* than *tshβa* basal transcript levels in cell cultures even in April, as suggested by the comparison of their respective qPCR quantification cycle values (Cq), is in agreement with our previous results by qPCR on whole pituitaries and previous observation by *in situ* hybridization (17). This large difference in transcript abundance is in agreement with the hypothesis (17) of a local role and retrograde action toward the brain of the *tshβb* paralog, as for PT-TSH in amniotes, in contrast to the endocrine role *via* the general blood circulation of the *tshβa* paralog, as for PD-TSH in amniotes.

### Stronger Inhibition by Thyroid Hormones of *tshβa* Than *tshβb* Expression in Atlantic Salmon

In salmonids, a rise of plasma T4 and/or T3 levels is observed at the time of smoltification [for reviews: (6, 45); Atlantic salmon, T4 and T3 (46); coho salmon *Oncorhynchus kisutch*, T4 (47, 48); coho, chinook salmon and steelhead trout, T4 and T3 (49); coho salmon, T4 and T3 (50, 51); masu salmon *Oncorhynchus masou*, T4 and T3 (52); masu salmon and amago salmon, *Oncorhynchus rhodurus*, T4 (53); masu salmon, T4 (54)]. As part of the thyrotropic axis, thyroid hormones (T3 and T4) are well-known to exert a negative feedback on the pituitary, downregulating TSH production in mammals and other vertebrates [for reviews: (9, 10)].

In line with this negative feedback, our study showed that thyroid hormones dose-dependently inhibited the expression of the transcripts of both salmon *tshβ* paralogs. Remarkably, the inhibitory effect was much stronger on *tshβa*, with up to more than 95% inhibition, than on *tshβb*, with a maximal inhibition of 25%.

For comparison, we measured the impact of thyroid hormones on *gh* transcript expression in Atlantic salmon pituitary cells. We also observed a moderate inhibitory effect of T4 and T3 on transcript expression, reaching a maximal inhibitory effect of 30%. Our previous studies in the eel also showed an inhibitory regulation of GH synthesis and release by thyroid hormones, exerted directly at the pituitary level (28), and which may represent an ancestral and largely conserved crosstalk between somatotropic and thyrotropic axes in vertebrates [for review: (55)].

In teleosts, before the availability of bioassays for directly quantifying TSH, one investigation mean was to look by histological methods at the activity of TSH cells. Baker thus observed that the addition of T4 to the culture medium of pituitary trout and eel culture prevents the hyperactivity of TSH cells *in vitro* [cytoplasmic degranulation (56); increase of uridine uptake (57)] suggesting that T4 has a direct negative effect on these cells. This cytological result was confirmed in guppy *Poecilia* *reticulata* (58, 59). *In vivo* experiments by Peter using implants of T4 in the pituitaries of goldfish and measuring radioactive iodine uptake by the thyroid gland also supported the existence of this negative feedback by T4 on the pituitary (60, 61). Later on, various *in vivo* studies in other teleosts confirmed these findings *via* direct measurements of *tshβ* mRNA levels using RNase protection assay [coho salmon (62)], Northern-blot [European eel (63); goldfish (64), turbot, *Psetta maxima* (65); red drum *Sciaenops ocellatus* (66)], or RT-PCR and qPCR [fathead minnow *Pimephales promelas* (67); bighead carp *Aristichthys nobilis* (68, 69); Japanese eel *Anguilla japonica* (70); red drum (71)]. Using *in vitro* primary culture of eel pituitary cells, Pradet-Balade and collaborators provided direct evidence that the negative feedback by T3 and T4 on *tshβ* mRNA levels was situated at the pituitary level (63).

All these former studies in teleosts, including salmonids, only concerned the “classical” *tshβa* paralog. In contrast, in our study, we also investigated the second paralog, *tshβb*, and showed that both T3 and T4 inhibited the transcript expression of the second paralog, but to a much lesser extent than for the “classical” *tshβa* paralog. We may suggest that the lower inhibitory effect of T3 and T4 on *tshβb* thus might not prevent the peak of *tshβb* transcript levels to happen during smoltification (17), despite an increase in plasma thyroid hormone concentrations at that time [Atlantic salmon, T4 and T3 (46)].

In the rat, Bockman and collaborators also reported a differential effect of thyroid hormones *in vivo* on the regulation of *tshβ* transcript levels in the PT (likely equivalent to salmon *tshβb*) and in the PD (likely equivalent to salmon *tshβa*) (72). T4 treatment did not affect the expression of *tshβ* in the PT, while it downregulated the expression of *tshβ* in the PD (72). The lack of effect of T4 on PT *tshβ* transcription could be related by the authors to the lack of detection of thyroid hormone receptor TR*β* mRNA in PT, while the receptor is expressed in the PD (72). However, other *in vivo* studies indicated that PT-TSH cells may be more sensitive to thyroid hormones during fetal development than in adulthood in rats. In fetal rats indeed, TSH-positive PT-specific cells react to changes in thyroid function similarly to PD thyrotropes: PT-TSH cell nuclei were enlarged in fetus from females treated with an antithyroid drug (PTU) and smaller in fetus from T4-treated females (73). In contrast, in adult rats, no ultrastructural alterations are observed after thyroidectomy (74) or treatment with PTU (75). As compared to mammals, thyroid hormone receptors have been duplicated through teleost and salmon specific whole genome duplications. Future studies may aim at determining which thyroid hormone receptor(s) are expressed by the two TSH pituitary cell types in Atlantic salmon and the regulation of their expression at the time of smoltification. This would also assess whether the actions of thyroid hormones are exerted directly or indirectly on each type of TSH cells.

### Stronger Inhibition by Cortisol of *tshβb* Than *tshβa* Expression in Atlantic Salmon

Cortisol, in most mammals and ray finned fish, and corticosterone, in most birds, amphibians, and reptiles, are the major glucocorticoids in vertebrates [for review: (76)]. In addition to its role in the control of metabolism and stress response in all vertebrates, cortisol is involved in osmoregulation in teleosts, and in particular in seawater adaptation of smolts in salmonids [for reviews: (7, 77)].

In our study, cortisol at the highest dose tested (10^-7^ M) was able to inhibit the mRNA levels of both *tshβ* paralogs, with a stronger effect on *tshβb* (about 70% inhibition) than *tshβa* (about 40% inhibition). To our knowledge, no study is available in teleosts concerning the effect of cortisol on TSH, even on the “classical” *tshβa* paralog. No effect was observed on *gh* transcript levels.

In human, it is known for a long time that glucocorticoid excess suppresses TSH secretion (78–80), while deficiency increases it (78, 81, 82). In rodents, early *in vivo* studies also reported a suppressive effect of glucocorticoids on basal TSH release (83–85). However, *in vitro* studies on rat pituitary cells in culture reported no changes in basal TSH levels after corticosterone treatment (86). In chicken, corticosterone inhibits *in vivo*, but not *in vitro*, sensitivity of thyrotropes to CRH at embryonic stage, suggesting an indirect mechanism of glucocorticoids on TSH in sauropsids (87).

In Atlantic salmon, plasma cortisol levels are low in winter and early spring (less than 10 ng/ml) and peak in May up to 40 to 100 ng/ml (88, 89), values largely compatible with the cortisol dose effective in our study. Same observation was made in coho salmon (90). The fact that cortisol strongly reduces *tshβb* mRNA levels in our study suggests that this hormone may potentially contribute to the termination of the *tshβb* peak observed at the end of April and May (17).

### Stimulation by CRH of *tshβa* But Not *tshβb* Expression, and No Effect of TRH on Both Paralogs in Atlantic Salmon

We compared the effects of CRH and TRH, both main central regulators of thyrotropic axis in vertebrates, on Atlantic salmon *tshβ* a and b paralog expression.

We showed a slight (about 1.5-fold increase), but significant, stimulatory effect of CRH on the transcript levels of *tshβa*, but not on those of *tshβb*. In the presence of T3 or cortisol, which downregulated the levels of both *tshβ* paralogs, CRH kept its stimulatory effect on *tshβa* mRNA levels, but still had no effect on *tshβb.* Furthermore, the effect of CRH on *tshβa* mRNA levels was largely enhanced in the presence of high inhibitory doses of T3, reaching up more than 15-fold increase (as compared to T3 alone), in the presence of 10^-8^ M T3. In contrast, TRH has no effect on either *tshβa* nor *tshβb* in the absence or presence of inhibitors. These results demonstrated a stimulatory effect of CRH specifically on *tshβa*, but not *tshβb*, paralog expression.

For comparison, we also analyzed the effects of CRH and TRH on *gh* transcript levels. CRH, but not TRH, had a slight but significant stimulatory effect on *gh* mRNA levels. Our previous studies reported a stimulatory effect of CRH on GH release in the European eel, which suggested a key role of CRH in the control of several neuroendocrine (corticotropic, somatotropic, and thyrotropic axes) (29).

In mammals, TRH was discovered for its stimulatory role on TSH secretion by the adenohypophysis (91). Concerning the PT, *in vivo* treatment in the rat showed a stimulatory effect of TRH on PD-*tshβ* transcript levels (likely equivalent to salmon *tshβa*), but no effect on PT-*tshβ* (likely equivalent to salmon *tshβb*) (72). The authors were able to relate this differential regulation to the expression of TRH receptor transcripts in the PD but not in the PT. Future studies may aim at investigating the expression of CRH receptor(s) in salmon pituitary *tshβa*- and *tshβb-*cells, taking into account that multiple CRH receptors are present in teleosts [for reviews: (92, 93)]. This would also allow to decipher the mechanisms of enhanced effect of CRH on *tshβa* expression in the presence of T3: whether basal *tshβa* expression in cell culture is almost maximal and needs to be inhibited for revealing potential stimulatory actions, as shown for instance for GH production by pituitary cell cultures in turbot (30); or whether T3 inhibits *tshβa* expression but also stimulates CRH receptor expression.

In teleosts, few studies have yet investigated the potential role of CRH on the thyrotropic axis. Two studies reported a stimulatory effect of CRH on thyrotropic axis, one *in vivo* on thyroid activity in goldfish (94) and one *in vitro* on TSH secretion by coho salmon pituitary cell culture (95). Another study reported no change of *tshβ* mRNA levels after CRH treatment in common carp (*Cyprinus carpio*) pituitary cell culture (96). Concerning TRH, it became apparent that in non-mammalian vertebrates, notably in teleosts, TRH not always possesses a TSH-releasing role [for reviews: (10, 11)]. The first studies reporting no effect of TRH on TSH release were performed *in vivo* and used indirect measurements. Wildmeister and Horster showed that administration of TRH did not induce exophthalmos in goldfish, while treatment with TSH did (97). In this species, treatment with TRH was indeed ineffective in affecting plasma T4 concentrations (98). In the African lungfish *Protopterus ethiopicus*, radioiodine uptake by the thyroid gland was not affected by TRH injection, but increased by TSH (99). However, other studies using the same kind of indirect measurements did show a stimulatory role of TRH [for review: (100)]. Bromage even described an inhibitory effect of TRH injection to guppies on thyroid and TSH cell activities (101). It is only later that *in vitro* experiments directly demonstrated species-specific differences in the role of TRH on TSH in teleosts. While TRH is able to increase *tshβ* mRNA levels in bighead carp (68, 69) and Japanese eel (70), it is not the case in common carp (96, 102). In coho salmon, CRH stimulated TSH secretion, while TRH had no effect (95).

CRH ability to stimulate TSH secretion in anuran amphibians was demonstrated by *in vitro* (103) and *in vivo* studies (104–106), and when comparison was made, a greater potency of CRH than TRH on TSH was observed [for reviews: (1, 11, 107)]. A stimulatory effect of CRH on TSH release was also found in chelonian sauropsids, as investigated in turtles (108, 109). In birds also, *in vivo* and *in vitro* studies reported a stimulatory effect of CRH on TSH (87, 110, 111).

CRH neurogenesis has been shown to increase from parr to early-smolts in anadromous Atlantic salmon (112). In landlocked salmons receiving thyroxine to augment thyroid hormone plasma levels to those of anadromous fish, the rate of CRH neurogenesis is elevated to anadromous fish levels (112). CRH has been shown to stimulate locomotor activity in juvenile salmon (113). The present study shows that CRH exerts a specific stimulatory effect on *tshβa* expression, but has no effect on *tshβb.* It thus demonstrates that neither CRH (nor TRH which has no effect on both tsh*β* paralogs) would be involved in the triggering of the remarkable peak of pituitary *tshβb* paralog expression occurring at smoltification (17).

### No Effect of SRIH on *tshβa* and *tshβb* Expression in Atlantic Salmon

Somatostatin (or somatotropin-releasing inhibitory hormone, SRIH) was discovered for its inhibitory role on GH release in mammals (114–116), a role well conserved in vertebrates including teleosts [for review: (55)]. In addition to the inhibition of GH, SRIH may also exert an inhibitory effect on basal or TRH-induced TSH secretion in mammals [rat (115); human (117)].

In our study, using primary culture of salmon pituitary cells, SRIH showed its classical inhibitory action on GH, inducing more than 70% inhibition of *gh* mRNA levels. In contrast, SRIH had no effect on both *tshβa* and *tshβb* mRNA levels.

In teleosts, early pioneer studies in goldfish addressed the potential inhibitory control of the thyrotropic axis. Pituitary transplantation (61), pituitary stalk sectioning, or hypothalamic lesioning (118) stimulated thyroid activity (measurement of conversion ratio, cell height, and radioiodine uptake). These studies provided indirect evidence for the existence of a brain TSH-inhibitory factor (TIF). Peter and McKeown (100) then investigated the potential role of SRIH as TIF in goldfish. They showed that injection of SRIH inhibited thyroid radioiodine uptake and suggested that SRIH action was mediated by inhibitory effect on TSH release. To our knowledge, no other studies investigated the potential role of SRIH on TSH in teleosts.

In birds, *in vivo* and *in vitro* studies showed an inhibitory effect of SRIH on TRH- or CRH-stimulation of TSH secretion (111, 119). This inhibition was likely mediated by SRIH receptors sstr2 and sstr5, which are expressed by the thyrotropes (120, 121). In amphibians, SRIH does not affect basal TSH secretion by bullfrog pituitary cells in culture (122).

### Specific Stimulation by IGF1 of *tshβb* Expression in Atlantic Salmon

IGF1 is an endocrine growth factor mainly produced by the liver under the control of pituitary GH, and acting on many target tissues to promote growth in mammals as well as in the other vertebrates [for review: (123)]. As a component of the somatotropic axis, IGF1 exerts a negative feedback on pituitary GH. IGF1, as well as GH itself, is also involved in the regulation of other functions such as reproduction, and osmoregulation in teleosts [for reviews: (124, 125)]. IGF1 has also been proposed as one important internal signal in the cross-talks between growth, metabolism and triggering of puberty in mammals as well as in teleosts [eel (32); for reviews: (124, 126)].

In salmonids, IGF1 circulating levels have been shown to steadily increase from before to during smoltification [Atlantic salmon (127, 128); chinook salmon (129); masu salmon (130); for reviews: (6, 45)], and IGF1, together with GH and cortisol, has been proposed to regulate in smolt the adaptation of gills to osmoregulation in seawater [for reviews: (131, 132)].

In the present study, we found a dose-dependent inhibitory effect of IGF1 on pituitary *gh* transcripts (up to more than 85% inhibition), in agreement with the conservation of IGF1 negative feedback on the somatotropic axis throughout vertebrates.

Strikingly, our study revealed that IGF1 dose-dependently stimulates *tshβb* transcript expression (up to 5-fold increase, according to the experiments). In contrast, no effect was observed on *tshβa* mRNA levels, indicating that IGF1 stimulatory effect is specifically exerted on the *tshβb* paralog.

To our knowledge, our study is the first to report a direct pituitary regulatory role of IGF1 on *tshβ* mRNA levels in any vertebrate. IGF1 receptors are expressed in the ovine PT (133) and IGF1 has been shown to regulate mitogen-activated protein kinase in primary cultures of ovine PT cells (134). Three IGF1-receptors paralogs have been identified in salmon (135). Future studies may investigate which of these receptors is expressed in the pituitary, and more specifically by cells expressing the *tshβb* paralog, to decipher whether the IGF1 stimulatory effect is exerted directly on *tshβb* cells.

We recently demonstrated that *tshβb* transcript levels peak during smoltification in Atlantic salmon in April concomitantly with rheotaxism inversion initiating downstream migration (17). Considering our present data on the specific stimulatory effect of IGF1 on this *tshβb* paralog, we can hypothesize that the increase of plasma IGF1 levels, which may start as early as February and lasts until May (127, 128), may be involved in the induction of smoltification-related *tshβb* expression.

In juvenile salmon, smoltification is preceded by a marked increase in body growth rate (130). As for puberty, where IGF1 is one of the internal cues linking body growth to activation of the gonadotropic axis, IGF1 may represent a key internal signal linking body growth to triggering of smoltification-related neuroendocrine changes in salmon.

In teleosts including salmons, as in other vertebrates, IGF1 is not only produced and released in the blood by the liver, as an endocrine factor, but also expressed locally in various tissues, such as gonad, gill, muscle, fat, heart, kidney, spleen, brain, and pituitary itself [for review: (136)], where it exerts local paracrine/autocrine actions. In tilapia, *igf1* transcript*s* are expressed in hypothalamic neurons with IGF1-immunoreactive axons projecting to the pituitary; *igf1* transcripts are also expressed by some pituitary cells including some gonadotropes, and the number of *igf1*-expressing gonadotropes increases at puberty as well as during seasonal reproduction (137). This led the authors to propose that pituitary IGF1 may act as paracrine/autocrine stimulator of gonadotropic cells during puberty and reproductive season [(137); for review: (124)]. We may suggest a similar role for IGF1 on *tshβb* thyrotropic expressing-cells during smoltification in salmon. Future studies will aim at investigating the localization and regulation of the *igf1* transcript in salmon brain and pituitary cells, during smoltification, to infer whether the stimulatory control on *tshβb* expression may be exerted by locally produced IGF1.

IGF1 has been proposed to play a key role in the mediation of vertebrate life-traits [for reviews: (138, 139)]. Our present findings suggest a role of IGF1 in the smoltification step during the life history of the long-river Loire-Allier salmon. This opens new research perspectives on the interactions between internal and environmental cues in the induction of smoltification in salmonids.

## Conclusion

In conclusion, this study provides the first data on the neuroendocrine factors involved in the differential regulation of the expression of the two *tshβ* paralogs in teleosts. Future studies, including localization of neurohormone and hormone receptors on different cell types of the salmon pituitary, should aim at investigating whether these regulations are exerted directly on *tshβa* or *tshβb*-cells, or indirectly *via* interactions with other pituitary cells. Thyroid hormones had a stronger inhibitory effect on *tshβa* than *tshβb* in salmon, similar to the differential regulation by thyroid hormones of *tshβ* from PD *versus* from PT in the rat. CRH stimulated the expression of *tshβa* with no effect on *tshβb*, which can also be compared to the stimulatory effect of TRH on *tshβ* from PD but not from PT, in the rat. This is the first report of IGF1 direct regulatory role on pituitary *tshβ* in any vertebrate species. Strikingly, IGF1 specifically stimulated the expression of *tshβb*, allowing us to infer a potential key role of IFG1 in the triggering of the *tshβb* peak at smoltification and crosstalk between body growth and smoltification metamorphosis.

## Data Availability Statement

The raw data supporting the conclusions of this article will be made available by the authors, without undue reservation.

## Ethics Statement

The animal study was reviewed and approved by Cuvier Ethic Commitee France.

## Author Contributions

SD, PM, KR: design of experiments. PM: supervision of fish husbandry. MF, GM, KR: cell culture. MF, GM, KR: qPCR. SD, KR: writing the manuscript. All authors: discussion of the results and reviewing the manuscript. All authors contributed to the article and approved the submitted version.

## Funding

This work was supported by grants from the French National Research Agency SALTEMP-BIOADAPT ANR-12-ADAP-0021-01 and from the European community, Innovative Training Network, ITN IMPRESS MSCA-ITN-2014-ETN N° 642893. MF was recipient of a PhD fellowship from ITN IMPRESS.

## Conflict of Interest

The authors declare that the research was conducted in the absence of any commercial or financial relationships that could be construed as a potential conflict of interest.

The reviewer PGV declared a past co-authorship with one of the authors SD, to the handling editor.
